# Sustainability of a mobile phone application-based data reporting system in Myanmar’s malaria elimination program: a qualitative study

**DOI:** 10.1186/s12911-021-01646-z

**Published:** 2021-10-18

**Authors:** Win Han Oo, Kaung Myat Thu, Julia C. Cutts, Win Htike, Kyawt Mon Win, May Chan Oo, Naw Hkawng Galau, Paul A. Agius, Pyae Phyo Htoon, Ellen Kearney, Aung Thi, Ye Win Aung, Min Min Thein, Kathryn Rosecrans, Kyi Tun Lwin, Aung Kyi Min, Freya J. I. Fowkes

**Affiliations:** 1 Disease Elimination Program, Burnet Institute, Melbourne, Australia; 2 Health Security Program, Burnet Institute Myanmar, Yangon, Myanmar; 3 National Malaria Control Program, Ministry of Health and Sports, Nay Pyi Taw, Myanmar; 4 Department of Epidemiology and Preventive Medicine, Monash University, Melbourne, Australia; 5 Melbourne School of Population and Global Health, University of Melbourne, Melbourne, Australia; 6 Save the Children International, Yangon, Myanmar; 7 Department of Infectious Diseases, The Peter Doherty Institute for Infection and Immunity, University of Melbourne, Melbourne, VIC Australia

**Keywords:** Malaria, Surveillance, eHealth, mHealth, Sustainability, Myanmar

## Abstract

**Background:**

Strengthening surveillance systems to collect near-real-time case-based data plays a fundamental role in achieving malaria elimination in the Greater Mekong Subregion (GMS). With the advanced and widespread use of digital technology, mHealth is increasingly taking a prominent role in malaria surveillance systems in GMS countries, including Myanmar. In Myanmar’s malaria elimination program, an mHealth system called Malaria Case-based Reporting (MCBR) has been applied for case-based reporting of malaria data by integrated community malaria volunteers (ICMVs). However, the sustainability of such mHealth systems in the context of existing malaria elimination programs in Myanmar is unknown.

**Methods:**

Focus group discussions were conducted with ICMVs and semi-structured in-depth interviews were conducted with malaria program stakeholders from Myanmar’s Ministry of Health and Sports and its malaria program implementing partners. Thematic (deductive followed by inductive) analysis was undertaken using a qualitative descriptive approach.

**Results:**

Technological and financial constraints such as inadequate internet access, software errors, and insufficient financial resources to support mobile phone-related costs have hampered users’ access to MCBR. Poor system integrity, unpredictable reporting outcomes, inadequate human resources for system management, and inefficient user support undermined the perceived quality of the system and user satisfaction, and hence its sustainability. Furthermore, multiple parallel systems with functions overlapping those of MCBR were in use.

**Conclusions:**

Despite its effectiveness and efficiency in malaria surveillance, the sustainability of nationwide implementation of MCBR is uncertain. To make it sustainable, stakeholders should deploy a dedicated human workforce with the necessary technical and technological capacities; secure sustainable, long-term funding for implementation of MCBR; find an alternative cost-effective plan for ensuring sustainable system access by ICMVs, such as using volunteer-owned mobile phones for reporting rather than supporting new mobile phones to them; and find a solution to the burden of multiple parallel systems.

***Trial registration*:**

Not applicable.

**Supplementary Information:**

The online version contains supplementary material available at 10.1186/s12911-021-01646-z.

## Background

The World Health Organization (WHO) defines mHealth as “a medical and public health practice supported by mobile devices, such as mobile phones, patient monitoring devices, personal digital assistants, and other wireless devices” [1]. With the increasingly widespread use of digital technology, mHealth is becoming more prominent across the world, including in developing countries [1, 2]. Mobile devices, especially mobile phones, are increasingly used in many healthcare programs for various preventive, curative, surveillance and research purposes, including malaria elimination programs [1–4].

Although the global burden of malaria, a mosquito-borne infectious disease, has reduced over the last two decades, approximately half of the global population is still at risk [5, 6]. In the Greater Mekong Sub-region (GMS, comprising Cambodia, China (Yunnan Province), Lao People’s Democratic Republic, Myanmar, Thailand and Vietnam), despite a 76% reduction in the reported number of malaria cases and a 95% reduction in malaria deaths to relatively low levels between 2010 and 2018 [5], malaria remains a public health challenge due to the complex epidemiology of malaria, and resistance of *P. falciparum* to artemisinin and other antimalarial medicines [7]. Considering this, in 2014 WHO’s Malaria Policy Advisory Committee recommended eliminating malaria from the GMS by 2030 [8].

To achieve malaria elimination, it is important to strengthen surveillance to ensure local health care professionals have easy access to real-time case-based reporting data in order to implement timely interventions [8, 9]. Recently, mobile network coverage, the numbers of mobile phone users and internet access through mobile phones have increased substantially in all GMS countries [10–12]. In this context, mHealth is expected to enable real-time case-based malaria surveillance.

**Fig. 1:**
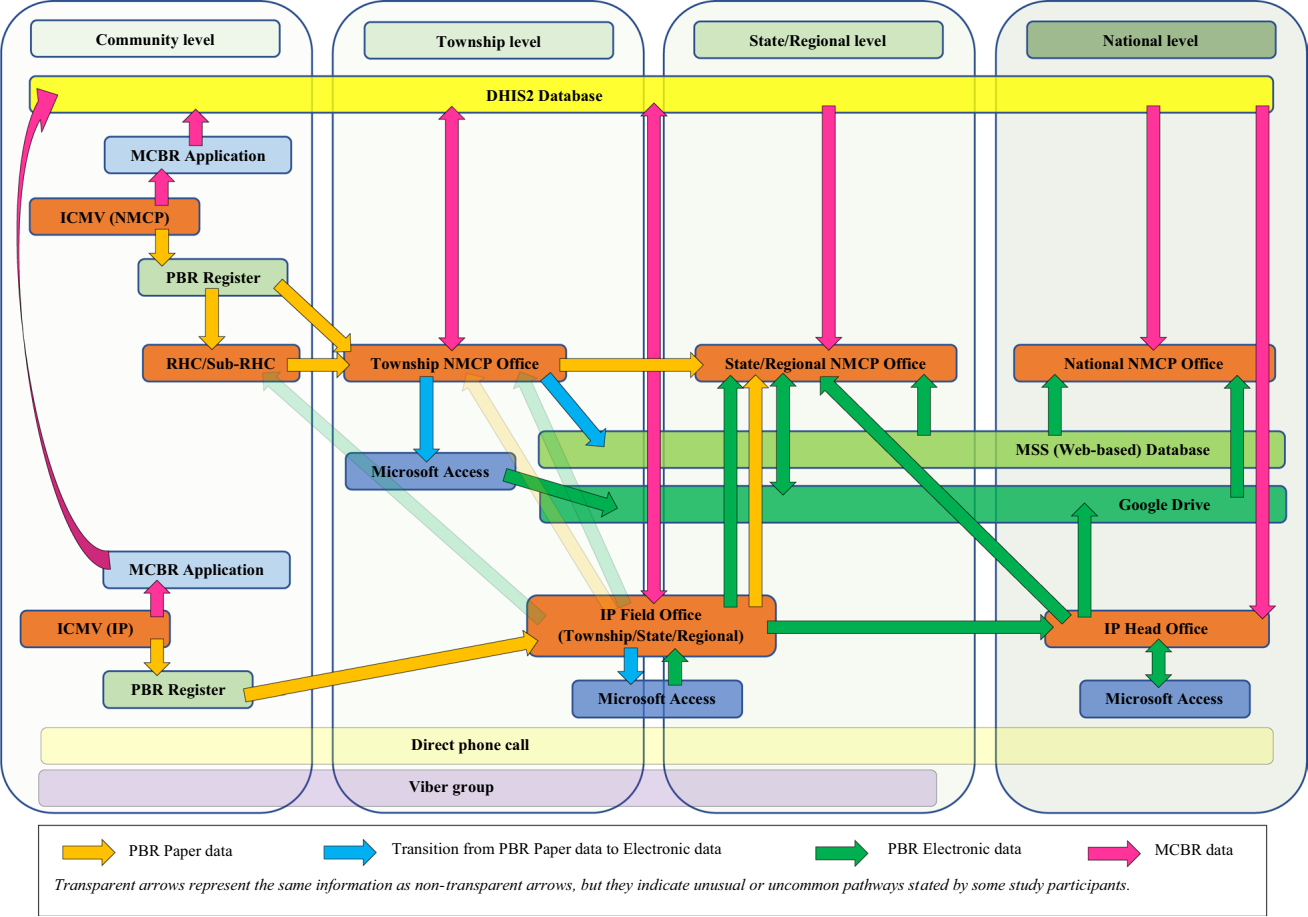
Flow of malaria case-based reporting data in the MCBR and PBR systems. *DHIS2* District Health Information System 2, *ICMV* Integrated Community Malaria Volunteer, *IP* Implementation partner, *MCBR* Malaria Case-based Reporting (system/application), *NMCP* National malaria Control Program, Myanmar, *MSS* Malaria Surveillance System, *PBR* Paper-based reporting, *RHC* Rural Health Centre; *Sub-RHC* Sub-Rural Health Centre. This figure was published in Win Han Oo et al. [13] and is used with permission of Win Han Oo, et al.

As in many GMS countries, malaria surveillance in Myanmar has traditionally relied on paper-based reporting (PBR). Malaria service providers in the community, including basic health staff of Myanmar’s Ministry of Health and Sports (MoHS) and integrated community malaria volunteers (ICMVs), supply free malaria prevention, diagnosis, treatment, and referral services in their communities. They record patient demographic information, malaria rapid diagnostic test (RDT) results and treatment information in a nationally standardized carbonless malaria register (Additional file 1). The data-filled register sheets are sent to or collected by ICMV supervisors at the township level, typically monthly. In township-level field offices, the paper-based data is entered manually into Microsoft Access, and the electronic data is forwarded to the higher levels by uploading onto Google Drive (Fig. 1) [13]. This lengthy chain of reporting through the PBR system may hinder timely and localised implementation of malaria elimination interventions [13].

To resolve the shortcomings of the PBR system in Myanmar’s malaria elimination program, Save the Children, an implementing partner, introduced a mobile phone application-based reporting system named Malaria Case-based Reporting (MCBR) for timely reporting of case-based data by ICMVs (Additional file 2). In MCBR, provided they are connected to the internet, malaria case-based data entered by ICMVs directly into the application on their mobile phones is instantly uploaded onto the dedicated District Health Information System 2 (DHIS2) database (an open-source software platform for reporting, analysis and dissemination of data for health programs, developed by the University of Oslo Health Information Systems Programme [14]) (Fig. 1). MCBR was piloted in 2017 and rolled out in early 2018, and by the end of the year more than 1,500 ICMVs managed by Myanmar’s National Malaria Control Programme (NMCP) and its implementing partners (IPs) were using it in 47 townships of 8 states/regions across Myanmar [13]. MCBR was initially found to be superior to the conventional PBR, because it enabled more accurate and complete data to be reported in a much timelier manner, and was favoured by ICMVs and their supervisors because of its efficiency [13]. However, challenges to its long-term sustainability (i.e., meeting current healthcare needs without compromising future utility [15]) and wide-scale national application need to be identified and resolved. Here we report findings of a qualitative assessment of the sustainability prospects of the MCBR system in the context of Myanmar’s malaria elimination program.

## Methods

**Fig. 2:**
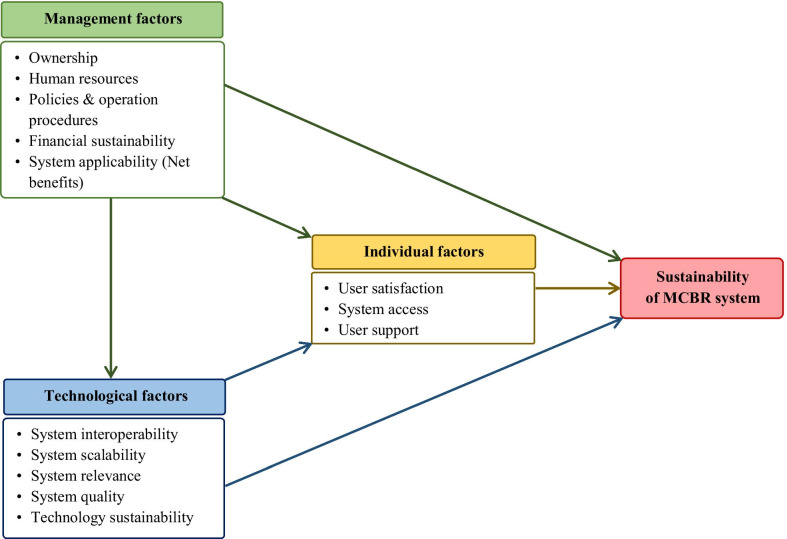
Thematic framework of factors influencing sustainability of the MCBR system. The authors developed this figure in Microsoft Office Word based on the information adapted from: Muhambe et al. [16]

This study was part of a larger study named “Assessing the effectiveness of the Malaria Case-Based Reporting (MCBR) application compared to the Paper-Based Reporting (PBR) system for the reporting of malaria cases in Myanmar: a mixed methods evaluation study”. The study protocol was approved by the Alfred Ethics Committee (273/19) (Additional file 3) and the Institutional Review Board 1, Myanmar Ministry of Health and Sports (IRB 1 / 2019 – 1) (Additional file 4), and all methods and procedures were performed in accordance with the relevant guidelines and regulations set by those institutions. Written informed consent was obtained from all participants. Findings on effectiveness, feasibility, utility, and cost-effectiveness of the MCBR system have been published elsewhere [13]. This paper evaluates the sustainability of the MCBR system by using the “parameters for evaluating sustainability of mHealth systems in developing countries” proposed in Muhambe et al. [16] whereby the factors influencing sustainability of mHealth systems were grouped into three broad themes—individual, technological and management factors, and relevant sub-themes as shown in Fig. 2.

The research team employed qualitative methods, including focus group discussions (FGDs) with 84 ICMVs in groups of 5–6 (total 14 FGDs) and semi-structured in-depth interviews with 14 malaria program stakeholders from MoHS and its IPs (total 14 interviews), to assess malaria situations, malaria-related services, and mechanisms of malaria data reporting (including PBR and MCBR systems) in their areas, facilitators of and barriers to using MCBR, and their perceptions of the applicability and sustainability of MCBR [13]. Purposive sampling with predetermined criteria for recruitment was used; the criteria included age, gender, locality, and malaria performance of the ICMVs, and type of organization (NMCP or IP), locality, designation/rank/roles, and level of representation of the malaria program stakeholders. Both male and female ICMVs were stratified by their performance defined by their average number of monthly RDT tests, and low and high performers were included in the FGDs equally. Malaria program staff at different levels of NMCP and its IPs, representing different geographical areas of Myanmar, were interviewed (Table 1). The FGDs and interviews were conducted in October 2019 to February 2020 and reflected the experiences of the ICMVs and stakeholders in 2018–19.

**Table 1:** Socio-demographic characteristics of the research participants, counts and per cent (%)

Characteristics	FGD	Interview
Numbered surveyed	83	14
Age (years)*		
≤ 30	32 (38.6)	*ND*
31–50	47 (56.6)	*ND*
≥ 51	4 (4.8)	*ND*
Sex		
Male	38 (45.8)	8 (57.1)
Female	45 (54.2)	6 (42.9)
Duration of malaria experience (years)^†^		
< 1	4 (7.0)	0
1–5	42 (73.7)	9 (62.3)
6–10	11 (19.3)	4 (28.6)
≥ 11		1 (7.1)
* ND*	4 (7.0)	
Position/designation		
ICMV	83 (100.0)	–
Field supervisors/data collectors	–	3 (21.4)
Data/monitoring and evaluation staff	–	3 (21.4)
Project manager/team leader	–	4 (28.6)
Regional officer/program manager/director	–	4 (28.6)
Level of representation		
Village/Village tract level	83 (100.0)	–
Township/district level	–	4 (28.6)
State/regional level	–	7 (50.0)
National level	–	3 (21.4)
Geographical attribute		
Kachin State	11 (13.3)	1 (7.1)
Kayin State	12 (14.5)	1 (7.1)
Mon State	36 (43.4)	3 (21.4)
Mandalay Region	12 (14.5)	1 (7.1)
Sagaing Region	12 (14.5)	2 (14.3)
Nay Pyi Taw Union Territory	0	1 (7.1)
Yangon Region	0	5 (35.7)
Representing organization		
NMCP	24 (28.9)	6 (42.9)
IP	59 (71.1)	8 (57.1)

*FGD* focus group discussion, *ICMV* integrated community malaria volunteer, *NMCP* Myanmar National Malaria Control Programme, *IP* implementation partner, *ND* not determined

^*^Age range of ICMVs: 18–60 years

^†^Malaria experience of interview participants: 2–16.3 years

The FGDs and interviews were conducted in person by trained facilitators or interviewers, with the help of facilitation/interview topic guides which were fine-tuned after pilot testing (Additional file 5). The sustainability of MCBR was approached in several ways, including direct open questions about its sustainability potential and factors influencing its sustainability. Interviews were conducted in private places in the primary language of the participants, with translators when necessary, and were audio-recorded with the consent of the participants. Transcripts of the discussions were later translated verbatim into English by the Myanmar-based research team. Data processing, management and analysis were assisted by NVivo version 12 (QSR International).

Thematic (deductive followed by inductive) analysis was undertaken using a qualitative descriptive approach. The data analysis process included data immersion, coding, and categorization (development of major themes and sub-themes) by two independent researchers (KMT and WHO), who then discussed the identified themes and subthemes to reach consensus. Emergent themes were also captured and incorporated into the thematic framework during analysis. The qualitative findings from FGDs and interviews were triangulated for comprehensive narrative synthesis [13].

## Results

As suggested by Muhambe et al*.* [16], qualitative findings on factors influencing the sustainability of the MCBR system were grouped into individual, technological and management factors, and each included sub-themes as described in Fig. 2. A summary of key findings on the factors and recommendations provided by study participants is given in Tables 2 and 3, and are discussed in detail below.

**Table 2:** Summary of key findings on the factors influencing the sustainability of the MCBR system

Major themes	Sub-themes	Findings
Individual factors	User satisfaction	Technological constraints make users unsatisfied Diverse ideas of user preference over PBR and MCBR systems
	System access	Technical problems with supported mobile phones Data managers lacking suitable computing devices for data access and management Financial and logistic burden of mobile phone maintenance Financial unsustainability of mobile phone-related costs, especially for national scale-up Insufficient mobile internet access Areas without internet access to be left out in national scale-up of MCBR
	User support	Poor IT literacy of the ICMVs Not enough training for ICMVs and stakeholders No proper user support system, especially for troubleshooting technological problems
Technological factors	System interoperability	Use DHIS2 platform endorsed by Myanmar MoHS
	System scalability	Financial and technological constraints for nationwide scale-up Basic health staff suggested as potential MCBR users
	System relevance	Data elements of MCBR reflect those of PBR Job-aid function helps ICMVs follow national malaria treatment guidelines Stock management module not covering all stocks Missing auto-alert system for positive case notification
	System quality	Simple, easy to use, and potentially timely Software bugs and errors, unsatisfactory system response time, unpredictable and unreliable outcomes Inconvenient data management Unsatisfactory output data quality
	Technology sustainability	Concerns with maintenance and improvement of mobile application (software)
Management factors	Ownership of the system	NMCP to take sole ownership of the system IPs thought NMCP is not currently ready to take over the system due to many constraints NMCP believes it is already in position to take over the system
	Human resources	Dropout of ICMVs trained for MCBR No separate focal person to manage MCBR at all levels in both NMCP and IP
	Policies and operational procedures	Lack of standard procedures and policies for proper operation of MCBR Mobile phone-based reporting not possible in non-government-controlled areas because of local security issues Concerns over standalone use of MCBR without physical documents for future reference
	Financial sustainability	Completely donor-funded currently Uncertainty about financial support after 2023
	Applicability of MCBR data	NMCP and IP do not apply MCBR data for practical applications, relying only on PBR data for such purposes Doubtful effectiveness of MCBR for malaria elimination due to many constraints, despite its potential

*DHIS2* District Health Information System 2, *ICMV* integrated community malaria volunteers, *IP* implementation partner, *IT* information technology, *MCBR* Malaria Case-based Reporting (system/application), *MoHS* Ministry of Health and Sports, Myanmar, *NMCP* National Malaria Control Program, Myanmar, *PBR* paper-based reporting

**Table 3:** Summary of recommendations for sustainability of the MCBR system as proposed by the study participants

Themes	Recommendations
General	Resolve all, or at least some, of the existing technical and operational challenges Develop and apply policies and standard operating procedures for proper operation of MCBR Develop a proper sustainability plan for MCBR
Better user support	Support better mobile phones for ICMVs, and computers or tablets for data managers Conduct additional MCBR training for all NMCP and IP stakeholders; conduct additional MCBR and mobile phone use trainings for ICMVs
Better workforce	Deploy a dedicated focal person for managing MCBR at each level Deploy a well-functioning user support system with an IT technician in each township or at least in each district, or empower township level staff with MCBR-related IT capacities
Technology sustainability	Approach Myanmar national experts to manage future upgrades, updates, and modification of the system
Financial sustainability	Apply alternative models for system access like “bring your own device” in which the MCBR application is installed on the ICMV’s own mobile phones Myanmar MoHS to secure funds for MCBR once (if) support from international donors ceases

*ICMV* integrated community malaria volunteers, *IP* implementation partner, *IT* information technology, *MCBR* Malaria Case-based Reporting (system/application), *MoHS* Ministry of Health and Sports, Myanmar, *NMCP* National Malaria Control Program, Myanmar, *PBR* paper-based reporting

### Individual factors

#### User satisfaction

Both ICMVs and stakeholders believed that MCBR would fulfil the purposes they want to achieve with malaria case reporting, especially regarding its timeliness, simplicity, ease of use, and requiring no physical transportation for reporting unlike PBR; in addition, they stated that their use of this modern technology improved their social status. However, they also reported many challenges in using MCBR, such as dependence on internet access, software errors, and data synchronization problems [13]. Among ICMVs, this induced anxiety around their work performance and that of the system, leading some users to feel unsatisfied.

Whilst many ICMVs wanted to continue using MCBR if the existing challenges are resolved, others reported that they preferred to use PBR. A few ICMVs reported they liked the idea of using PBR and MCBR in combination, whereas others disagreed because of the added work it created.

Similarly, none of the MoHS and implementing partner stakeholders wanted to discontinue either the MCBR or PBR systems, although they reported technological and operational issues in using them. Although some stakeholders expressed a strong preference for MCBR, provided that the aforementioned challenges are resolved, some stakeholders expressed a preference for PBR, reasoning that it is the most efficient reporting system in the current context.

PBR system is the most efficient one.

PBR has been used for a long time and familiar with both ICMVs and higher levels [stakeholders].

PBR has solid documents and is auditable.

PBR system is [the only] convenient channel.

PBR is the only available and possible method to use in their area.

(NMCP and IP stakeholders)

Although there were diverse ideas on the standalone use of the PBR and MCBR systems, some stakeholders acknowledged that the use of both systems during the transition to an all-electronic mHealth system in the future may be necessary.

#### System access

Since the roll-out of MCBR, the NMCP and IPs have provided the ICMVs with Samsung Galaxy J1 or Samsung J2 mobile phones with a SIM card and the MCBR application already loaded. According to ICMVs, they were supplied with mobile credit of between 5,000 MMK (~ 3.5 USD) per three months and 10,000 MMK (~ 7.0 USD) per month. However, the ICMVs raised several complaints about the phones, such as small screens, batteries draining too quickly, and poor performance which worsens with time; it was claimed these problems hampered their MCBR reporting considerably.

An NMCP stakeholder reported that data managers who access and manage the data reported through the MCBR system are not equipped with suitable computing devices, such as laptop computers or mobile tablets. Currently, they access and manage large amounts of MCBR data through mobile phones, which is impractical.

There are, let’s say, hundreds of blood tests reported from one township. So, it is not an easy task to access all these data using a mobile phone. (Project manager level stakeholder, IP)

Moreover, ICMVs working in the field expressed concern regarding potential loss or damage of the mobile phones, which they did not consider to be their own property, and many ICMVs reported losing or damaging their mobiles. They called for protective accessories like phone cases and waterproof bags.

Stakeholders expressed their concern about the sustainability of supporting mobile phones costs, including mobile handsets, phone credits and maintenance fees. They claimed that the cost is high, and it burdens the supporting organizations in the long run, particularly when the MCBR system is scaled up nationally.

The office had prepared an additional ten percent stock of mobile phones as a buffer. But we have already consumed this buffer stock and no more lost or damaged phones could be replaced. (Field supervisor level stakeholder, IP)

With the intention of ensuring sustainability, many stakeholders proposed other models like *“bring your own device”* where the MCBR application would be installed on the ICMVs’ own mobile phones (if compatible), potentially freeing up budget to support mobile phone credits. Some IP stakeholders reported that they already used this model when they were unable to support any more mobile phones for the ICMVs for MCBR.

As more and more volunteers have their own phones, volunteers use the app [MCBR] in their own phones. (Program manager level stakeholder, IP)

Another major concern for MCBR sustainability raised by the ICMVs and stakeholders was mobile network coverage. Despite significant expansion of the network in Myanmar during 2014–16, many ICMVs claimed that insufficient mobile internet access made timely reporting via MBCR impractical, and resulted in additional costs for the ICMVs to travel to places with internet access.

I cannot upload the data because mobile internet signal is poor in my village. I need to go to the place with a better internet access. (ICMV, Kayin State)

Mobile internet access problems were identified by ICMVs and stakeholders as a main contributor to data synchronization problems and poor data access in the MCBR system. One stakeholder reported that although MCBR was largely implemented in a remote area, it was not successful because of limitations in mobile internet access. Some stakeholders pointed out that even if the MCBR were expanded nationwide, some ICMVs in remote areas would be unable to use MCBR because of unavailability of mobile networks. Therefore, MCBR can be expected to operate in these areas only after the establishment of a stable mobile internet network.

In addition, electricity is not available from the national power grid in some remote villages; their main power sources are solar panels and generators. ICMVs requested support for power banks and solar panels, especially those living in remote areas.

#### User support

Another major barrier to MCBR use is the poor information technology (IT) literacy of the ICMVs. Some had never used a smartphone before. All MCBR users received training on the MCBR application, including some basics of mobile phone usage. However, some ICMVs and stakeholders reported that the training they received was inadequate. They wanted additional training, such as refresher training on MCBR, training on mobile phone use for ICMVs, and specific training for supervisors, data managers and monitoring and evaluation staff of NMCP and IPs.

The first contact point of the ICMVs for IT troubleshooting is their peer ICMVs and their immediate supervisors, who are usually township level staff, although some ICMVs contact regional-level staff for troubleshooting. The regional-level stakeholders preferred that ICMV troubleshooting of IT issues occurred first at the township level. They voiced the need to build the capacity of field supervisors to solve MCBR-related IT and data management issues. Moreover, it was also pointed out that, for transformation into a well-functioning eHealth system, including for proper management of the MCBR system, at least one IT technician should be deployed in each township, or at least in each district in addition to existing staff.

We need to do capacity building to township (malaria) focal persons to upgrade their skills. If we can do so, it would be better for MCBR. (Regional officer level stakeholder, NMCP)

### Technological factors

#### System interoperability

Regarding the potential integration of reporting other volunteer-serviced diseases, such as dengue, filariasis, tuberculosis, leprosy, and HIV/sexually transmitted infections, into MCBR, some ICMVs expressed their willingness for such an integration. However, some ICMVs negated the idea for many reasons such as their poor IT literacy, poor internet coverage in their residing areas and added work burden. Many stakeholders were also willing to do the integration however they also expressed concerns about the existing unresolved challenges of MCBR and compatibility of MCBR with reporting systems currently being used in other disease control programs. Nevertheless, one program manager-level stakeholder from an IP noted that DHIS2 is an MoHS-endorsed platform for malaria (through MCBR) and other diseases, which creates potential for the MCBR system to interconnect with other disease control programs. Therefore, better understanding of the existing eHealth architecture and collaboration among the different public health programs will make the integration possible.

One of the good things about MCBR … it’s basically DHIS-2, which is the chosen platform of the Ministry of Health. So, I think that is a really big plus, because all of the data is going into a system that will be sort of standardized in the way that the ministry wants it. (Program manager level stakeholder, IP)

#### System scalability

Most stakeholders claimed that it was possible to scale-up the MCBR system nationally, and some even showed their eagerness for it. However, stakeholders had concerns about the financial and technological constraints which could be exaggerated with national scale-up.

MCBR still has problems in field implementation. … If we nationally scale up the MCBR, the situation would be worsened in some townships. … So, we better solve current problems and upgrade the current application into a stable one. Then we may think about the national scale-up. (Team leader, NMCP)

When considering expansion of MCBR to other users, many of the stakeholders mentioned basic health staff such as Midwives and Public Health Supervisors-2, whose geographical coverage and consultation numbers are generally higher than those of the ICMVs. Nevertheless, some stakeholders were afraid the introduction of MCBR would be an additional burden to the basic health staff, who are already busy with many tasks. Conversely, some stakeholders think using MCBR will reduce the burden of their paperwork.

#### System relevance

The data elements and job aid function of MCBR aligned with the standardized carbonless malaria register and national treatment guidelines, respectively. Both ICMVs and stakeholders reported that it was useful for the ICMVs to follow the national malaria treatment guidelines, and that the data reported through MCBR were in line with the existing data requirements. However, the ICMVs wanted the stock module of the application to include all types of supported stocks, such as paracetamol tablets, multivitamin tablets, and oral rehydration salt packs, in addition to the antimalarial medicines and malaria RDTs.

Many stakeholders suggested that supervisors are not constantly monitoring the DHIS2 database to recognize when a malaria positive case is reported, and called for an auto-alert system in the MCBR, enabling responsible supervisors to be notified directly (e.g., by SMS) when an ICMV reports a positive case through the MCBR application. The lack of such an alert system was criticised as a weakness in reaching the goal of real-time notification and reporting of malaria cases. Currently, MCBR users call their supervisors on the telephone to provide urgent notification of malaria cases.

#### System quality

Although the ICMVs appreciate the MCBR application for its simplicity and ease of use, many complained that delivery outcomes of their report in MCBR were unpredictable and unreliable, and they sometimes need to confirm delivery status by other methods such as direct phone calling. The stakeholders also reported that the quality of the MCBR data in the DHIS2 database was unsatisfactory [13], undermining its further applicability for program implementation. However, many of the stakeholders expected data quality to improve.

#### Technology sustainability

Although none of the stakeholders expressed concerns about the technological sustainability of the DHIS2 platform, many higher-level stakeholders were concerned about potential dependence on international software developers for technical assistance, including maintenance and further improvement of the MCBR application. In the long run, such dependence would be burdensome for organizations supporting the use of MCBR; the problem could be resolved if Myanmar national experts could manage the system.

### Management factors

#### Ownership of the system

The stakeholders perceived that everyone working with the MCBR system at different levels—including ICMVs, NMCP and IP staff, policymakers and software developers—shares responsibility for its sustainability. However, all NMCP and IP stakeholders agreed that the NMCP should ultimately have sole ownership of the system. On inquiring about the readiness of NMCP to take overall ownership of the MCBR system, some NMCP stakeholders reported that they were confident to take over MCBR because NMCP had set up its own server, recruited the required number of personnel and trained them properly.

So, with our current conditions, it is possible (for the NMCP to take over the MCBR ownership). First, the server is ready. Next, the manpower. In the townships where the MCBR is operating, we have already appointed data focal persons such as data assistant. Team leaders and regional officers have received trainings for MCBR. (Team leader, NMCP)

Nevertheless, IP stakeholders suggested that NMCP ownership is currently impossible, and that the NMCP would require more time to completely take over MCBR given current human resources, technical, technological, and financial limitations of NMCP, although its capacity is improving.

I definitely think it's possible [that NMCP totally takes over MCBR]. Like I said, it's just about making sure the requirements [for managing MCBR] are known and that they're ready to take over. … I think we need to do it carefully in a planned way, in a phased manner, which is what we're trying to do. (Program manager level stakeholder, IP)

#### Human resources

Although some NMCP stakeholders reported they had the required human resources, other NMCP and IP stakeholders reported human resource problems regarding the MCBR system, such as attrition of trained ICMVs, and inadequate number and capacity of personnel at the management level. NMCP and IPs lack dedicated focal persons for managing and monitoring the MCBR system at all levels. Instead, the focal person for the PBR system is responsible for managing the MCBR system, including monitoring, supervision and providing feedback to all respective ICMVs. In the NMCP, some of the ICMV supervisors have to take responsibility for other vector-borne disease control activities. They cannot focus on MCBR during the rainy season, because they need to work on outbreaks of other infectious diseases, such as dengue.

If we can have a separate person for this purpose [for monitoring the MCBR system], it would be better. … Now the existing staff here have to do these [MCBR-related] tasks. In the dengue season, the malaria positivity is also high. Then, we all get crazy. (Regional officer level stakeholder, NMCP)

Both NMCP and IP stakeholders pointed out that a dedicated focal person for managing MCBR is necessary to maintain the database and to make sure everything is really working in the MCBR system. The NMCP stakeholders stated that, for NMCP, it is preferable that this focal person be an internal staff member rather than a seconded staff member, and ideally should be posted at the township level.

The best situation is having an IT focal person in each volunteer-occupied township. … That’s why I prefer to train our own staff who are young and have familiarity with IT. (Regional officer level stakeholder, NMCP)

#### Policies and operational procedures

Some participants highlighted the lack of standard procedures and policies to guide the proper operation of the MCBR system. Identifying the entity responsible for the cost of replacing or repairing a lost or damaged phone was also a common problem among the managing stakeholders. Without an agreed-on policy, the stakeholders had to balance the ICMVs’ careless use of MCBR phones against their reluctance to use the devices because of the potential financial burden on them. Currently, this problem is solved in various ways, such as ICMVs paying for phones they lose or damage, all peer ICMVs sharing the cost of new phones for ICMVs who lose or damage them, or, most frequently, the managing organizations paying for replacement or repair (creating a financial and logistical burden).

Another barrier to the geographical scaling up of the MCBR system is that armed forces who manage non-government-controlled areas reject implementation of the MCBR system in their areas because of local security issues associated with the use of mobile phone GPS capability. Despite these areas being high malaria transmission areas with many ongoing interventions, MCBR cannot be used to report these activities.

Although the ICMVs and stakeholders had diverse ideas about totally replacing the PBR system with MCBR, both expressed concern about using MCBR for malaria reporting without the physical documents associated with the PBR system and the loss of this reference source for data quality validation and donor audits.

#### Financial sustainability

Currently, MCBR is almost completely funded by an international donor agency, including the costs for its development, hosting and maintaining the server, procurement of mobile phones, and supporting volunteers with phone credits. Some stakeholders expressed concern about the sustainability of MCBR after 2023, when they believe funding may cease. Although some funding gaps are foreseen, stakeholders hope that MoHS will allocate some funds to ensure future sustainability of the MCBR system. However, a proper sustainability plan for the MCBR system is yet to be developed.

… I know that the Union Minister [of MoHS] is very supportive of our electronic health information system. So, I hope that the ministry will be able to find some funds for sustaining the operation of MCBR in the future. (Program manager level stakeholder, IP)

#### Applicability of MCBR system (net benefits)

All stakeholders reported that, for the time being, they could not apply the MCBR data from the DHIS2 database for their decision-making, resource allocation or program management due to poor perceived data quality [13].

On asking about the potential usefulness of the MCBR system to the malaria elimination program, some ICMVs reported that MCBR is an effective reporting method for malaria elimination, but some disagreed, mentioning factors such as the lack of timely positive case notification. Many of the stakeholders expected MCBR will become a better surveillance system for malaria elimination than PBR if current obstacles and constraints are resolved.

## Discussion

The evaluation study on the medium-term performance, challenges and cost-effectiveness of the MCBR system has shown that MCBR is an effective and efficient system for malaria surveillance in the context of malaria elimination programs by providing timely and accurate case-based data [13]. However, this research identified many technical and operational challenges in its operation that threaten its sustainability. On evaluating the prospects for the sustainability of the MCBR system, many modifiable and non-modifiable intrinsic and extrinsic factors come into play, mostly technological, financial, and operational barriers affecting the operation and reliability of the system. MCBR users were satisfied with the performance of the system, but they do not recommend using it alone in the malaria elimination program because of its limitations, such as dependence on internet access, software errors, data synchronization problems, small phone screens and draining phone batteries too quickly. In addition, the IT literacy of ICMVs is poor, and IT technicians who could solve these issues are not deployed at township level. Moreover, ICMVs working in the field expressed concern about potential loss and damage of the mobile phones which, they believe, are not their own property. However, supporting ICMVs with mobile phone-related costs burdens the NMCP and IP organizations, and funding support for MCBR by international donor agencies beyond 2023 is uncertain. Therefore, national scale-up of MCBR to all ICMVs and to other health care providers, such as basic health staff, would be challenging. Technical assistance from international software developers for maintenance and further improvement of the MCBR application, preferably from Myanmar in-country software developers, would be required. To establish a well-functioning sustainable mHealth system in Myanmar for control and elimination of infectious diseases, including malaria, everyone working in the health system at different levels, including ICMVs, NMCP and IP staff, policymakers and software developers, must share responsibility and ownership.

User satisfaction and preference for a tool over alternatives are important for ensuring continuous use of the tool and therefore its sustainability [16]. Assessment of user preference regarding MCBR and PBR systems revealed that the study participants favoured the MCBR mainly because of its capacity for timely electronic reporting of malaria cases, which is not possible with the PBR system alone. Conversely, they do not trust the MCBR system completely, for several reasons, so do not want to give up the PBR system and totally replace it with MCBR. Their future faith in using MCBR depends on the ability of stakeholders to resolve its existing and upcoming challenges.

The most challenging issue for the sustainability of the MCBR system was found to be maintaining the ICMVs’ system access by overcoming technical difficulties. For a mobile internet-based reporting system like MCBR, stable access to the internet is essential. Like in other developing countries [16, 17], problems with mobile internet access were found to be the most challenging issues with MCBR. Therefore, the MCBR system will be impossible to implement in some remote areas of the country until the network is improved. These areas will surely be left out of a nationwide scale-up, and alternative solutions for malaria reporting must be identified for areas lacking reliable internet access so that local malaria case data can incorporated into the same system. Internet access problems, like problems in data synchronization, can reduce the reliability of the system, undermining the users’ trust in system quality in the long term. If left unresolved, they can lead to demotivation of the users and discontinuation of system use [16]. Similarly, solving the technical difficulties encountered by data managers in accessing and validating MCBR data in the DHIS2 database, investigation of users’ needs, improvements in the data structure and tools, and tailored trainings for data managers would be beneficial.

Technology is constantly changing; the MCBR system, including its mobile application, will also require upgrades, updates, and modification for many reasons in the future. In contrast, DHIS2, a free and open-source platform, has been field-tested for more than 15 years, demonstrating its scalability, interoperability and compatibility with a wide range of mobile devices [14], so seems a good option for a donor-dependent project in a developing country. Hiring foreign software developers for further modification and maintenance of the MCBR application seems impractical in the long term. Developing in-country technical experts is an alternative, more sustainable option.

Another constraint for the sustainability of the MCBR system is the lack of clarity about sustained financial support. As with mHealth projects in other donor-supported countries [16], the MCBR project is donor-dependent, and its fate after 2023 is unknown. Currently, the NMCP and its implementing partners provide all ICMVs with mobile phones and cover service-related costs, which constitutes the largest proportion of costs for implementing the MCBR surveillance system [13].Therefore, the financial burden of national scale-up cannot be managed under the current model, and NMCP and its implementing partners should test solutions, such as the *“bring your own device”* model, to reduce costs. However, acquiring and owning compatible devices in remote villages is challenging in Myanmar and many other settings [17].

Even if a relevant and reliable MCBR system with sustainable financial support is achieved, qualified human resources and a good support system are needed to ensure the system operates sustainably. Some staff in NMCP and IP organizations were found to be engaged in many tasks other than managing the MCBR; a dedicated focal person for proper operation and management of the MCBR system, at least at the township level, would be beneficial. Moreover, user support in the MCBR system seems to be insufficient and ad hoc. MCBR users at different levels need more capacity building; creation of a continuous learning environment with frequent regular or refresher trainings would benefit them in the long run. Deployment of proficient monitoring and evaluation staff with IT capacity in each township will be advantageous for monitoring reports, analysing data, and advising the program; providing training to program and field staff and ICMVs to improve their capacity; and troubleshooting MCBR-related problems encountered at both community and management levels. Only with a good user support system will effective use of the MCBR and user satisfaction be achieved and hence its sustainability be ensured. However, to address the human resource gaps for efficient system operation and proper user support would require a programmatic evaluation of the human resources. The program stakeholders will need to determine what are the existing and required human resources, how the gaps can be filled, and how much the available budget allows to deploy additional human resources.

The sustainability of the MCBR system is also influenced by the introduction of alternative electronic data reporting systems with similar capabilities. Design and deployment of newer systems or tools should allow interoperability with existing systems to avoid duplication of function and inefficiencies [16]. The MCBR system was developed to reflect the existing PBR system with the intention of replacement, therefore the two systems have overlapping functions. However, in late 2019, the NMCP pilot-tested a new internet-based system for malaria case-based electronic data entry and electronic data sharing and reporting called the Malaria Surveillance System (MSS) (Web-based) in three regions of Myanmar. In MSS (Web-based), malaria data from paper registers is entered directly into a web-based system which is hosted on an in-country data server (Fig. 1). During the Workshop on Surveillance and Malaria Database Management (September 23–25, 2020), the NMCP announced that the electronic data entry and reporting component of the PBR system, previously done using Microsoft Access and Google Drive, will be totally replaced by the MSS (Web-based) nationwide in early 2021, first in the NMCP implementing areas, then in IP areas. However, the transition has been delayed due to ongoing COVID-19 pandemic and political factors.

At the same time, the MCBR application was modified and upgraded into the Malaria Case Based Reporting and Surveillance (MCBRS) application, based on the same DHIS2 platform. In the MCBRS system, functions for recording and reporting of malaria case investigation, foci investigation and response activities will be added to existing malaria case testing and treatment functionality, and the application is intended to be used by both ICMVs and basic health staff. The stock management module was dropped in MCBRS. An email-based alert system for positive case notification will also be incorporated into MCBRS. It will solve two weaknesses of MCBR, although the effectiveness and efficiency of these new functions in the existing context still needs to be evaluated. Currently, addition of the functions for malaria case investigation, foci investigation and response activities and the email-based alert system is still on process. However, the transition from MCBR to MCBRS occurred during 2020, and by the end of the year, only MCBRS was left in use in both NMCP and IP implementation areas (personal communication with the staff from Save the Children International in 2021).

Finally, both the PBR with MSS (Web-based) and MCBRS systems will remain in operation for malaria case-based reporting; merging the two ongoing systems, with overlapping functions and different database platforms, is bound to be difficult. The merge will require considerable interdepartmental cooperation and many technical and operational discussions. If one system is designated as the primary system, the sustainability of the other will depend on its successful integration with the first. Whichever system is chosen to be the primary system, either of the electronic or paper forms must be auditable because WHO’s certification process for malaria elimination needs proper and auditable documentation of malaria surveillance and case registration [9].

Upon a personal communication with Myanmar NMCP in 2021 regarding their way forward with the two systems, the NMCP has not made the ultimate decision on choosing a single reporting platform. They wanted to retain the MSS (Web-based) as the main electronic recording and reporting method of the PBR system, and the PBR will be continued at health facility levels. If the upgraded MCRBS system functions well, the PBR system will be replaced with it at the ICMV level.

Not only it is important to have a single integrated data reporting system in the malaria elimination program itself, but it is also important to integrate various mHealth interventions in various diseases control programs of the country. Otherwise, a proliferation of short-lived unconnected diverse digital tools will result in inefficiency with added workload and financial burden to the primary health care program, and ultimately the effectiveness and sustainability of primary health care interventions will be compromised [18]. MCBR, built on the MoHS-endorsed DHIS2 platform, has the potential to be integrated with other disease control programs in Myanmar as most of them use the same DHIS2 platform. However, the integration will require commitment of the MoHS and effective interdepartmental cooperation together with deeper understanding of existing eHealth architecture and programmatic evaluation of different disease reporting systems. Currently, different community-based health interventions are implemented in an integrated fashion, and the ICMVs themselves are providing integrated services for many diseases other than malaria. Therefore, development of an integrated data reporting system of different primary health care programs will be necessary in the pathway to development of Universal Health Care in Myanmar and other GMS countries where the goal is to achieve Universal Health Coverage by 2030 [19].

The sustainability and growth of a system largely depends on the organization that has ownership of the system. Among the three endgame models of mHealth applications, namely government adoption, commercial adoption and hybrid model [20], MCBR aims at government adoption. In Myanmar, NMCP, the government leading body of malaria elimination, will assume ownership for the entire MCBR system in a few years, but must fill the existing health system gaps to improve and sustain its implementation. A well-defined organogram with dedicated technically empowered focal persons for the MCBR system; policies, guidelines, and standard operating procedures necessary for smooth and systematic operation of the system; and a sustainability plan with a promising funding source for the MCBR system, and ultimately for the development of a full-blown electronic malaria reporting system, is needed. Moreover, NMCP must find a way to streamline malaria reporting to remove the burden of multiple systems on the users. Afterall, scaling up and sustainability of mHealth applications is a dynamic process that should be continuously assessed and improved according to the changing circumstances, and the sustainability plan should apply a systems approach that harmoniously addressed all interconnected sectors such as the MAPS Toolkit of WHO [20].

Although the MCBR application has potential benefits as an effective, efficient and cost-effective electronic system for malaria case reporting by ICMVs [13], discussion with the study participants revealed that despite easy access to electronic data, both IP and NMCP stakeholders have not found the MCBR to be better than “the PBR system plus direct phone calling”. These findings related largely to the lack of timely case notification, system reliability and data applicability in the current environment.

Although the positive case notification function, and functions to record and report case investigation, focus investigation and response activities are being incorporated into the new MCBRS, the system needs to find a way to tackle two more issues related to malaria case-based reporting in the elimination setting. Firstly, the MCBR system and the iterated MCBRS report malaria cases diagnosed by RDT, mostly clinical malaria cases. They do not automatically count subclinical malaria cases, which are remnant infected cases that must be accounted for in the elimination program. Ensuring both clinical and sub-clinical cases detected by molecular malaria surveillance, such as polymerase chain reaction testing (when available), are readily accessible in the DHIS2 database will be beneficial for elimination planning and decision-making. Secondly, the MCBR system does not resolve difficulties around tracking and reporting of *Plasmodium vivax* malaria cases for relapse. Incorporating new functions such as reporting of the primaquine treatment completion status of the patient would be beneficial.

### Strengths and limitations of the study

This study included a variety of participants from both NMCP and IPs, covering all levels of the malaria elimination program and a wide geographical distribution. Except for two malaria stakeholders from the national NMCP office (who could not be included as participants because they were study investigators), the sample can be said to be representative of users and stakeholders involved in the MCBR system. This study was conducted from late 2019 to early 2020, before some changes to the PBR and MCBR systems. Therefore, the study could not give detailed information about the new iterated MCBRS system, including its mobile phone application, and the MSS (Web-based) system, although some participants mentioned their initiation processes. However, some such information was included in this paper in order to give a full picture of the systems, especially in terms of sustainability prospects.

## Conclusions

Resolving the existing technological barriers will improve the reliability of the MCBR system, increasing the trust of its users and fostering its applicability. Stakeholders should deploy a dedicated workforce equipped with the necessary devices and technical capacities to efficiently run the MCBR system, as well as find a way to reduce the burden of multiple parallel systems. Improved confidence of the users in the system, supported with empowered human resources and adequate funding, will ensure the sustainability of the MCBR system. Although improving mobile internet coverage or the political situation are beyond the influence of the health sector, interdepartmental cooperative effort for simultaneous multisectoral improvement in line with the Sustainable Development Goals will also improve the applicability and sustainability of a promising mHealth intervention such as the MCBR. Unlike other mHealth interventions implemented in Myanmar, the rapid upgrading of the MCBR into MCBRS highlights its ability to be sustainable. Findings from this study will contribute to the sustainable design and implementation of further mHealth interventions in Myanmar, and the GMS more broadly. Sustainable implementation of appropriate, effective, efficient and integrated mHealth interventions will enhance the coverage and quality of health practices and services on the way towards achieving the goal of universal health coverage by 2030.

## Supplementary Information

**Additional file 1.** Standardized carbonless malaria register.

**Additional file 2.** Screenshots of the Malaria Case-based Reporting (MCBR) mobile application.

**Additional file 3.** Ethics approval certificate from Alfred Ethics Committee.

**Additional file 4.** Ethics approval certificate from IRB (1) of Myanmar MoHS.

**Additional file 5.** Topic guides for focus group discussions and interviews.

## Data Availability

The data, both audio files or transcripts of the interview and discussion sessions, cannot be made publicly available because it would breach compliance with the ethical framework of the Institutional Review Board 1, Myanmar Ministry of Health and Sports. De-identified individual participant data can be available from the data custodians only with the approval of the Chairman of the Institutional Review Board. Contact information of the chairman and data custodians are provided below: Chairman, The Institutional Review Board (1), Office Number (4), Ministry of Health and Sports, Nay Pyi Taw, Myanmar; (+95) 067 3431071, Email: info@mohs.gov.mm. Professor Freya Fowkes, Deputy Program Director (Maternal, Child and Adolescent Health), Head (Malaria and Infectious Disease Epidemiology), NHMRC Fellow, Burnet Institute, 85 Commercial Rd, Melbourne, VIC 3004, Australia, Email: freya.fowkes@burnet.edu.au. Dr. Win Han Oo, Senior Program Manager (Health Security), Burnet Institute, No. 226, 4th Floor, Wizaya Plaza, U Wisara Road, Bahan Township, 11201, Yangon, Myanmar, Email: winhan.oo@burnet.edu.au.

## Abbreviations

DHIS2: District Health Information System 2
FGD: Focused group discussion
GMS: Greater Mekong Sub-region
ICMV: Integrated Community Malaria Volunteer
IP: Implementation partner
IT: Information technology
MCBR: Malaria Case-based Reporting (System/Application)
MCBRS: Malaria Case-based Reporting and Surveillance (System/Application)
MoHS: Ministry of Health and Sports, Myanmar
MSS: Malaria Surveillance System
NMCP: National malaria Control Program, Myanmar
PBR: Paper-based reporting
RDT: Rapid diagnostic test (for malaria)
WHO: World Health Organization
