# Supplementary material for: Sustainability of a mobile phone application-based data reporting system in Myanmar’s malaria elimination program: a qualitative study
Source: BMC Med Inform Decis Mak. 2021 Oct 18;21:285. doi: 10.1186/s12911-021-01646-z (PMC8521268; doi:10.1186/s12911-021-01646-z)
Supplement: Supplementary file 1 — Additional file 1. Standardized carbonless malaria register. [file 12911_2021_1646_MOESM1_ESM.pdf]

Malaria case register (for volunteers)

State/Region: Township:tt Station/Rural Health Center: Sub-RHC:

Organization: Village: Name of volunteer: Reporting month & year:

Sr.	Date	Name	Age	Address	Sex		Pregnant	RDT result				Status		Antimalaria given						Referral to health facility	Malaria death	Seek treatment		History of traveling (within 2-4 weeks)	Occupation<br>1. Rubber plantation<br>2. Agriculture<br>3. Construction<br>4. Forestry<br>5. Mining<br>6. Others	Remark
								Positive			RDT negative															
					M	F		Pf	Pv	Mix		Not severe	Severe	ACT 24	ACT 18	ACT 12	ACT 6	Chloroquine	Primaquine			Within 24 hours	After 24 hours			
Total																										

Month	Particular	Malaria RDT	ACT 24	ACT 18	ACT 12	ACT 6	Chloroquine	Primaquine
	Remaining from previous month							
	Received for this month							
	Total							
	This month usage							
	Closing balance							

Signature:
Name:
Village:
Organization:
