# Supplementary material for: Sustainability of a mobile phone application-based data reporting system in Myanmar’s malaria elimination program: a qualitative study
Source: BMC Med Inform Decis Mak. 2021 Oct 18;21:285. doi: 10.1186/s12911-021-01646-z (PMC8521268; doi:10.1186/s12911-021-01646-z)
Supplement: Supplementary file 2 — Additional file 2. Screenshots of the Malaria Case-based Reporting (MCBR) mobile application. [file 12911_2021_1646_MOESM2_ESM.pdf]

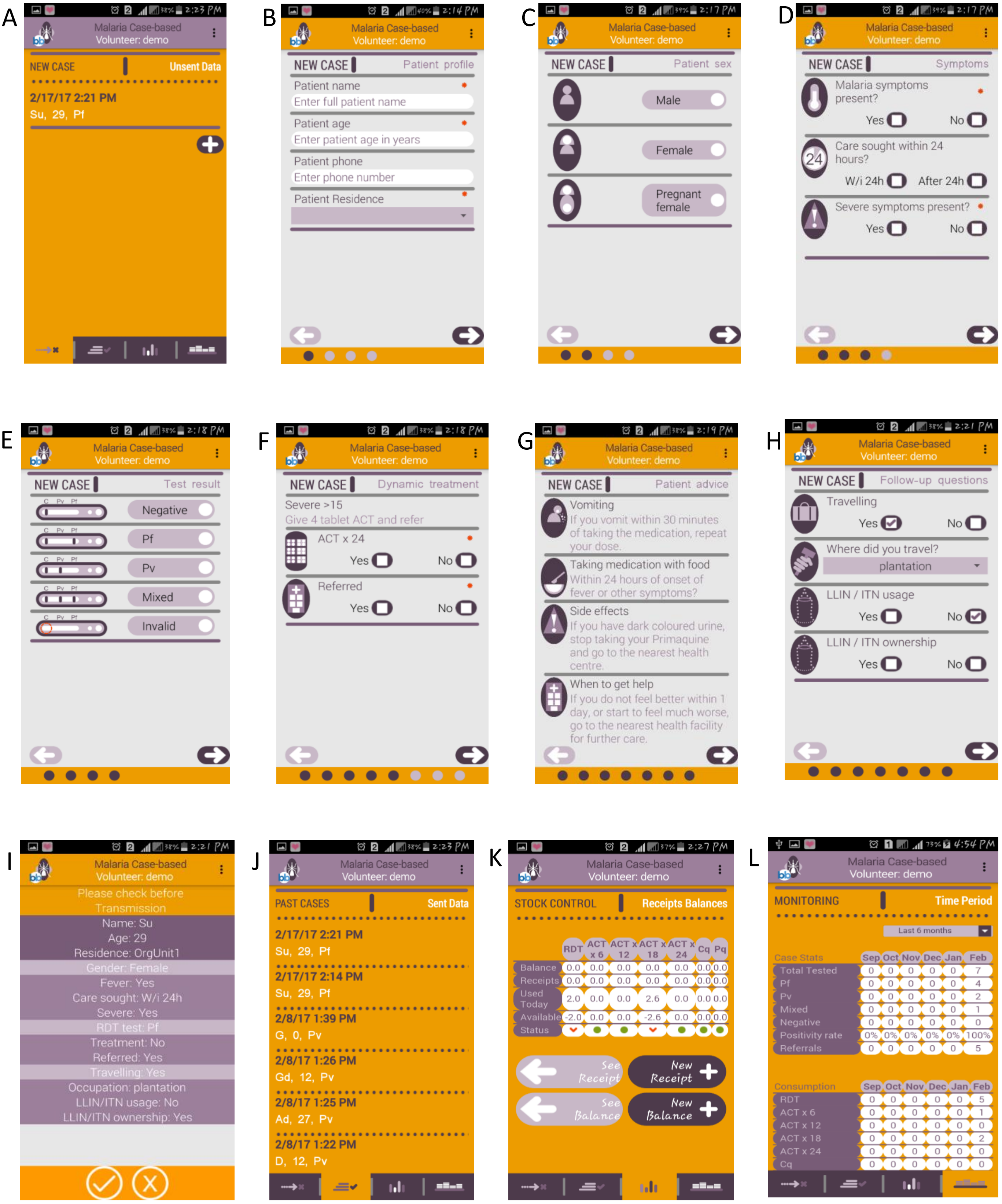

Figure 1 (A-L). Screenshots of Malaria Case-based Reporting (MCBR) mobile application. This figure was published in Win Han Oo, et al. A mobile phone application for malaria case-based reporting to advance malaria surveillance in Myanmar: a mixed methods evaluation. Malaria Journal. 2021;20(1): 167. and is used with permission of Win Han Oo, et al.
