# Supplementary material for: Sustainability of a mobile phone application-based data reporting system in Myanmar’s malaria elimination program: a qualitative study
Source: BMC Med Inform Decis Mak. 2021 Oct 18;21:285. doi: 10.1186/s12911-021-01646-z (PMC8521268; doi:10.1186/s12911-021-01646-z)
Supplement: Supplementary file 3 — Additional file 3. Ethics approval certificate from Alfred Ethics Committee. [file 12911_2021_1646_MOESM3_ESM.pdf]

ETHICS COMMITTEE CERTIFICATE OF APPROVAL

This is to certify that

Project No: 273/19

Project Title: Assessing the effectiveness of Malaria Case-Based Reporting (MCBR) compared to Paper-Based Reporting (PBR) for the reporting of malaria cases in Myanmar: a mixed methods evaluation study

Principal Researcher: A/Professor Freya Fowkes

Protocol Version 1.0 **dated:** 8-May-2019

Appendix 1: PIS - Focus Group Discussion with Integrated Community Malaria Volunteers version 2.0 dated: 17-May-2019
Appendix 1: PIS - Field observations with Integrated Community Malaria Volunteers version 2.0 dated: 17-May-2019
Appendix 1: PIS - Field observations with Implementing Partner stakeholders version 2.0 dated: 17-May-2019
Appendix 1: PIS - Field observations with Ministry of Health and Sports stakeholders version 2.0 dated: 17-May-2019
Appendix 1: PIS - In-depth Interviews with Implementing Partner stakeholders version 2.0 dated: 17-May-2019
Appendix 1: PIS - In-depth Interviews with Ministry of Health and Sports stakeholders version 2.0 dated: 17-May-2019
Appendix 1: PIS - Key Informant Interviews with Ministry of Health and Sports stakeholders version 2.0 dated: 17-May-2019
Appendix 1: PIS - Survey with Integrated Community Malaria Volunteers version 2.0 dated: 17-May-2019
Appendix 2: PICF - Focus Group Discussion with Integrated Community Malaria Volunteers version 2.0 dated: 17-May-2019
Appendix 2: PICF - Field observations with Integrated Community Malaria Volunteers version 2.0 dated: 17-May-2019
Appendix 2: PICF - Field observations with Implementing Partner stakeholders version 2.0 dated: 17-May-2019
Appendix 2: PICF - Field observations with Ministry of Health and Sports stakeholders version 2.0 dated: 17-May-2019
Appendix 2: PICF - In-depth Interviews with Implementing Partner stakeholders version 2.0 dated: 17-May-2019
Appendix 2: PICF - In-depth Interviews with Ministry of Health and Sports stakeholders version 2.0 dated: 17-May-2019
Appendix 2: PICF - Key Informant Interviews with Ministry of Health and Sports stakeholders version 2.0 dated: 17-May-2019
Appendix 2: PICF - Survey with Integrated Community Malaria Volunteers version 2.0 dated: 17-May-2019
Appendix 3: Research Timeline
Appendix 4: Focus Group Discussion Guide - ICMVs version 1.0 dated: 8-May-2019
Appendix 5: In-depth Interview Guide - Implementing Partners version 1.0 dated: 8-May-2019
Appendix 6: Key Informant Interview Guide - Ministry of Health and Sport version 1.0 dated: 8-May-2019
Appendix 7: Fieldwork Observation Guide - ICMVs version 1.0 dated: 8-May-2019
Appendix 8: Fieldwork Observation Guide - Stakeholders version 1.0 dated: 8-May-2019
Appendix 9: Survey - ICMVs version 1.0 dated: 8-May-2019

was considered by the Ethics Committee on ***30-May-2019****, meets the requirements of the National Statement on Ethical Conduct in Human Research (2007) and was* ***APPROVED*** *on* ***4-Jun-2019***

It is the Principal Researcher's responsibility to ensure that all researchers associated with this project are aware of the conditions of approval and which documents have been approved.

The Principal Researcher is required to notify the Secretary of the Ethics Committee, via amendment or progress report, of

- Any significant change to the project and the reason for that change, including an indication of ethical implications (if any);
- Serious adverse effects on participants and the action taken to address those effects;
- Any other unforeseen events or unexpected developments that merit notification;
- The inability of the Principal Researcher to continue in that role, or any other change in research personnel involved in the project;
- Any expiry of the insurance coverage provided with respect to sponsored clinical trials and proof of re-insurance;
- A delay of more than 12 months in the commencement of the project; and,
- Termination or closure of the project.

Additionally, the Principal Researcher is required to submit

- A Progress Report on the anniversary of approval and on completion of the project.

The Ethics Committee may conduct an audit at any time.

All research subject to the Alfred Hospital Ethics Committee review must be conducted in accordance with the National Statement on Ethical Conduct in Human Research (2007).

The Alfred Hospital Ethics Committee is a properly constituted Human Research Ethics Committee in accordance with the National Statement on Ethical Conduct in Human Research (2007).

SPECIAL CONDITIONS

The project should not commence until all approvals from other ethical review bodies have been granted, and copies of those approvals and any changes requested by those review bodies have been provided to the Alfred Hospital Ethics Committee.

CONSENT WAIVER

In accordance with the Office of the Health Services Commissioner's Statutory Guidelines on Research issued for the purposes of Health Privacy Principles 1.1(e)(iii) & 2.2(g)(iii), the Alfred Hospital Ethics Committee granted a consent waiver for the collection, use and disclosure of participants' health and personal information (as detailed in the Victorian Specific Module dated 8-May-2019).

SIGNED:

Professor John J. McNeil
Chair, Ethics Committee

Please quote project number and title in all correspondence
