# Supplementary material for: Sustainability of a mobile phone application-based data reporting system in Myanmar’s malaria elimination program: a qualitative study
Source: BMC Med Inform Decis Mak. 2021 Oct 18;21:285. doi: 10.1186/s12911-021-01646-z (PMC8521268; doi:10.1186/s12911-021-01646-z)
Supplement: Supplementary file 4 — Additional file 4. Ethics approval certificate from IRB (1) of Myanmar MoHS. [file 12911_2021_1646_MOESM4_ESM.pdf]

The Government of the Republic of the Union of Myanmar

Ministry of Health and Sports

Institutional Review Board - 1

Office No (4), NayPyiTaw

Certificate of Approval

IRB number:	2019-001
Approval number:	IRB 1/2019-1
Date of Approval:	30.9.2019 (valid up to 1 calendar year)
Project title:	Assessing the effectiveness of Malaria Case-based Reporting (MCBR) application compared to Paper-Based Reporting (PBR) system for for the reporting of malaria cases in Myanmar: a mixed methods evaluation study
Principal Investigators:	Assoc. Prof. Freya Fowkes, Burnet Institute<br>Dr. Kyawt Mon Win, Assistant Director (Malaria)<br>Department of Public Health

Document accepted:

1. Ethical proposal
2. Full proposal (Version no. 2)
3. Proposal Summary (Version no. 2)
4. Agreement to comply with ethical guideline (Version no. 1)
5. Case Report Form (Version no. 2)
6. Informed consent (Version no. 2)
7. Study area :
8. Investigator CV
9. Annexes such as Questionnaires

Institutional Review Board, Department of Medical Research, Ministry of Health and Sports approves to conduct the proposed research project as it is in full compliance with the Declaration of Helsinki, Council for International Organizations of Medical Sciences guidelines and International Conference on Harmonisation in Good Clinical Practice guidelines.

Principal Investigator should be aware that there might be site monitoring visits at any time from IRB team during project implementation and should provide full cooperation to the team.

Dr. Myo Khin

Chairperson

Institutional Review Board–1

Ministry of Health and Sports

CC : Director General, Department of Medical Research

Assistant Permanent Secretary, International Relations Division, Ministry of Health and Sports

Approval is subject to following conditions:

- The principal investigator is required to notify the Secretary of the Ethics Committee, via amendment or progress report, of
  1) Any significant change (such as study area) to the project and the reason for that change, including an indication of ethical implications (if any);
  2) Serious adverse effects on participants and the action taken to address those effects;
  3) Any other unforeseen events or unexpected developments that merit notification;
  4) The inability of the Principal Investigator to continue in that role, or any other change in research personnel involved in the project;
  5) Termination or closure of the project

- The principal investigator for submitting the progress report at least 6 weeks prior to the expiry of the approved date to allow adequate time for the ERC for substantive and meaningful review and for assuring that the research is not conducted beyond the approved date.
- 6 months report should be submitted.
- Final report is to be provided to ERC at the end of the study.

Back page- informed consent, Clinic report form (for clinical/ if any)

Material Transfer Agreement and Data Transfer Agreement (if any)
