# Supplementary material for: Sustainability of a mobile phone application-based data reporting system in Myanmar’s malaria elimination program: a qualitative study
Source: BMC Med Inform Decis Mak. 2021 Oct 18;21:285. doi: 10.1186/s12911-021-01646-z (PMC8521268; doi:10.1186/s12911-021-01646-z)
Supplement: Supplementary file 5 — Additional file 5. Topic guides for focus group discussions and interviews. [file 12911_2021_1646_MOESM5_ESM.pdf]

Assessing the effectiveness of the Malaria Case-Based Reporting (MCBR) application compared to the Paper-Based Reporting (PBR) system for the reporting of malaria cases in Myanmar: a mixed methods evaluation study

Topic Guides for Focus Group Discussion and Semi-structured Interviews

The following are the topic guides for the focus group discussion with the ICMVs who reported their malaria case-based data through the MCBR and PBR systems and semi-structured interveiws with the stakeholders who are responsible for managing the PBR and MCBR systems at different levels in different geographical and organiation backgrounds.

The following topic guides were dedicated for the accessment of all evaluation aspects of the whole project. Questions for evlaution of the sustainablity prospects of the MCBR system are included specific parts of the topic guides.

Topic Guid for Focus Group Discussion Guide with ICMVs

Is it ok to audio record this conversation? a. Yes b. No

Responsible persons	Responsibility
Facilitator (F)	Lead the overall process and facilitate discussion to obtain enriched data using an ethical approach
Note taker (NT)	Note-taking, audio recording and supplementary facilitation
Translator	Translation of facilitator and participants discussion where necessary.

Background Information		
0.1	Name of F:	
0.2	Name of NT:	
0.3	Name of Translator:	
0.4	Date (DD/MM/YYYY):	
0.5	Start time:	
0.6	End time:	
0.7	Location (venue):	
0.8	Archival code	
0.9	Sex of participants (group)	
0.10	Age range of participants	

Themes, questions, and probes

No	Themes, questions, and probes
1.	Current malaria situation
	❖ What is the burden of malaria in your working village/area?<br>▪ Is it increasing or decreasing in your areas?<br>❖ How was it impacted on your village/community?<br>❖ How did you manage to help the malaria patients?<br>❖ Do you think malaria is still a priority health issue in your village/community?<br>▪ Why do you think so?

2.	Malaria control measures and available malaria provider(s) in the respective community
	❖ What are the available services/interventions for malaria in your community?<br>▪ Do you think are they enough to combat malaria?<br>▪ If not, what interventions are you going to suggest?<br>❖ Where does your community get the malaria interventions/services recently? Who are the malaria service provider(s)? Is it only you? Are there more malaria service providers?<br>▪ Do you think this/these providers fulfil the malaria service gap in the community?<br>▪ If not, why and what's your suggestion to meet the needs?<br>▪ Are they also overlapping each other in the malaria service provision?
3.	Malaria elimination
	❖ Have you heard 'malaria elimination'?<br>➢ If yes, what does it mean to you?<br>➢ What do you think your role will be in the malaria elimination program?
4.	Reporting of malaria cases
	❖ Can you tell me your experience of the reporting of malaria cases since you have been working as a volunteer? Please give examples.<br>➢ How have you reported the cases?<br>➢ Only by paper based reporting?<br>▪ If so, what are the barriers and enablers of PBR in the past? Please give examples.<br>❖ Have you ever noticed any barriers and/or enablers to timely reporting of malaria cases in the community currently?<br>▪ What are they? Please give examples.<br>▪ How do we enhance the enablers in the future? Please give examples.<br>▪ How do we overcome the barriers in the future? Please give examples.<br>▪ What kinds of support are necessary to overcome the barriers and from whom?
5.	Stock management
	❖ As a volunteer, can you explore experience of stock management?<br>❖ How did you manage? Are there any problems in particular, working as an ICMV?<br>❖ How has the carbonless paper helped (or not) in your stock management? Please give examples.<br>❖ How can we overcome the stock management issues and which type of supports are needed, and from whom?<br>❖ Any suggestions for future ICMV stock management?
6.	General views and perspectives on the current PBR and MCBR
	❖ Are you still willing to continue using PBR?<br>➢ If so, why?<br>❖ Do you know MCBR? Please describe it.

	➢ What is the difference between MCBR compared to PBR? Please give examples.<br>❖ Do you think MCBR is an effective system for reporting malaria cases<br>▪ Why? And How? Please give examples.<br>▪ What are the enablers of using MCBR application?<br>❖ Do you intended to regularly use MCBR instead of PBR?<br>▪ If yes, what influences your decision to use MCBR over PBR<br>❖ What are the specific barriers and enablers of using MCBR? Please give examples.<br>➢ Ease of use<br>➢ Data lost<br>➢ Sync error<br>➢ Bill for the use of online reporting<br>➢ If yes, why?<br>➢ If not, why?
7.	MCBR application
	❖ How do you think the MCBR could be improved in future?<br>❖ What features and/or functions do you want to add or remove into/from MCBR? Please explain one by one.<br>❖ How can we best put MCBR application in the context of ICMV?<br>❖ Do you think MCBR should extend to reporting of other ICMV diseases? Why?<br>❖ If we expand MCBR for reporting of other diseases, what kind of supports for ICMV will be needed? And from whom? Why?
8.	Training and support for MCBR
	❖ To what extent were you satisfied with the training you received on how to use the MCBR?<br>❖ What further training would be of benefit.<br>❖ What supports should be provided to volunteers if the volunteers have to use only MCBR for reporting. Please explain detail.<br>➢ Financial, in kind and/or moral supports
9.	Overall
	❖ Overall, do you think PBR can be replaced by MCBR for reporting of malaria cases<br>➢ Why do you think so?

This is the end of my questions. Do you have anything else you would like to say about this research?

Do you have any questions for me?

Thank you very much for your participation.

End of session

Topic Guide for Interview with Higher/Middle Level Program Staff

Is it ok to audio record this conversation? a. Yes b. No

**Background Information**		
0.1.	Name of interviewer:	______________________
0.2.	Date (DD/MM/YYYY):	_________
0.3.	Start time:	_________
0.4.	End time:	_________
0.5.	Job level (indicate):	Higher level/ Middle level policy maker
0.6.	Organization (indicate):	SCI/HPA/IOM/ NMCP
0.7.	How long have you worked in your current position (in years)?	_________
0.8.	How many years of work experience in malaria sector do you have (in total)?	_________

**Section 1: Roles and responsibilities**		
	Describe your current role and key responsibilities	
**Section 2: Current malaria situation**		
	What's the situation of malaria in your assigned territory?	
	Why do you think that the burden of malaria in your area is going up/down or plateauing?	
**Section 3: Reporting in malaria program**		
	Which reporting systems are being used for malaria cases and malaria interventions from ICMVs? (PBR, MCBR, ph call, social media, viber, etc.)	
	Can you please elaborate pros and cons of using each reporting system for the program? (Pros and Cons)	
	Which reporting system do you think is the most effective?	
	Do you think it is possible for this system to be scaled-up for use in Myanmar?	
**Section 4: Views and perspectives on paper-based reporting (PBR)**		
	Can you describe the paper-based reporting system for malaria case reporting?	
	Do you think PBR is an effective method of malaria surveillance in an elimination setting? Why? If not, why?	
	How do you receive and access the data collected by ICMVs using PBR?	

	How do you analyse the data collected by PBR?	
	What are barriers/challenges you experience in using PBR?	
	How do you think we can mitigate these barriers?	
	What barriers/challenges do you experience in implementing/managing the PBR reporting system?	
	How do you think we can mitigate these barriers?	
	What barriers/challenges do you think ICMVs using PBR would encounter?	
	How do you think we can mitigate these barriers?	
	What are some of the facilitators that enable ICMVs to use PBR?	
	What are reasons for why you are using PBR systems compared to other systems such as MCBR?	
	What are the advantages to using in PBR systems compared to MCBR?<br>For Case Detection vs. Reporting	
	Are you happy with the data quality of the PBR systems?<br>Why/ Why not?	
**Section 5: Views and perspectives on MCBR**		
	Can you describe the MCBR system for malaria case reporting?	
	Do you think MCBR is an effective method of malaria surveillance in an elimination setting? Why? If not, why?	
	How do you receive and access the data collected by VHVs using MCBR?	
	How do you analyse the data collected by MCBR?	
	What are barriers/challenges you experience in using MCBR?	
	How do you think we can mitigate these barriers?	
	What barriers/challenges do you experience in implementing/managing the MCBR reporting system?	
	How do you think we can mitigate these barriers?	
	What barriers/challenges do you think VHVs using the MCBR would encounter?	
	How do you think we can mitigate these barriers?	

	What are some of the facilitators that enable VHVs to use MCBR?	
	What are reasons for why you (MoH/IP Stakeholders) are using MCBR systems compared to other to systems such as PBR?	
	What are the advantages to using in MCBR systems compared to PBR?<br>For Case Detection vs. Reporting	
	What is the most useful part of the MCBR system? Why?	
	To what extent does the MCBR improve your access to reporting data?	
	Are you happy with the data quality of the MCBR systems?<br>Why/ Why not?	
**Section 6: Usability of MCBR**		
	How do you think the MCBR system could be improved?	
	How do you currently use the data from MCBR/DHIS2 to inform decision making around funding/resources allocation?	
	What is the most challenging part of accessing and/or using data gathered through MCBR/ DHIS2? Why?	
	What type of capability could be added to the MCBR software to help you with your work?	
	What concerns do you have in using/scaling up MCBR?	
**Section 7: Supports needed for applying MCBR in malaria program**		
	Which supports are needed for ICMVs to expand the MCBR as a routine and effective reporting channel?<br>(technical and training, financial, technological, political, advocacy, administrative, moral etc)<br>Are they been fulfilled?<br>If not, do you see any plan to fulfil those requirements?<br>If not, how can we best fulfil those requirements in time?	
	Which supports are needed for data assistants, M&E staff and/or any data staff who have responsibility to compile, enter and analyse data from MCBR? (technical and training, financial,	

	technological, political, advocacy, administrative, moral etc)<br>Are they been fulfilled?<br>If not, do you see any plan to fulfil those requirements?<br>If not, how can we best fulfil those requirements in time?	
	Which supports are needed for policy makers and/or decision makers to access and utilize data from MCBR? (technical and training, financial, technological, political, advocacy, administrative, moral etc)<br>Are they been fulfilled?<br>If not, do you see any plan to fulfil those requirements?<br>If not, how can we best fulfil those requirements in time?	
**Section 8: Sustainability in the context of ICMV**		
	Do you think MCBR application is sustainable in the context of ICMV if necessary improvements are being made and/or necessary supports are provided? Why? Why not?	
	What are the key factors for the sustainability of MCBR in reporting in malaria program? How do we address them to sustain the MCBR in long run?	
	Do you think NMCP will be able to maintainthe operation of MCBR without support from Save the Children? Why? Please discuss detail including the following discussion points:<br>Server maintenance, server expansion, safeguarding data.	
	What kinds of adaptations need to be made to the application and overall system to enable NMCP to maintain operation of MCBR?	
	What kinds of resources are needed to enable NMCP to maintain operation of MCBR?	
	Can we use app-based reporting channel for reporting of non-malaria ICMV diseases?	
	What kinds of adaptations need to be made to the application and overall system to enable reporting of non-malaria disease covered by ICMV?	

	What kind of resources are needed to enable reporting of non-malaria disease covered by ICMV?	
	Who will be using the app-based reporting channel (BHS, Midwife, etc.)? Please discuss.	
	What kinds of adaptations need to be made to the application and overall system to enable non-ICMV personnel (e.g., BHS, midwives) to use the application?	
	What kind of resources are needed to enable non-ICMV personnel (e.g., BHS, midwives) to use the application?	
**Section 9: Overall**		
	Do you think PBR can be totally replaced by MCBR for reporting of malaria cases in Myanmar malaria elimination program? Why /Why not?<br>If yes, how can we best transition from PBR to MCBR? What are the factors that need to be taken care off in the transition?	
	Do you have any other suggestions/ comments regarding the reporting of malaria cases? If yes, please make comments.	

Do you have any questions for me?

Thank you very much for your participation.

End of session

Topic Guide for Interview with National/State/Regional Level Policy Makers

Is it ok to audio record this conversation? a. Yes b. No

**Background Information**		
0.1.	Name of interviewer:	______________________
0.2.	Date (DD/MM/YYYY):	_________
0.3.	Start time:	_________
0.4.	End time:	_________
0.5.	Job level (indicate):	Higher level / Middle level<br>National/State/Regional policy maker
0.6.	Organization (optional)	______________________
0.7.	How long have you worked in your current position (in terms of year)?	_________
0.8.	How many years of work experience in malaria sector do you have (in total)?	_________

**Section 1: Roles and responsibilities**		
	Describe your current role and key responsibilities	
**Section 2: Current malaria situation**		
	What's the situation of malaria in your assigned territory?	
	Why do you think that the burden of malaria in your area is going up/down or plateauing?	
**Section 3: Views and perspectives on paper-based reporting (PBR)**		
	Can you describe the PBR system for malaria case reporting?	
	How do you receive and access the data collected by PBR? Please give examples.	
	How do you analyse the data collected by PBR?	
	Do you think PBR is an effective method of malaria surveillance in an elimination setting like Myanmar? Why? If not, why?	
	What are barriers/challenges in managing the PBR systems?	
	What are the advantages to using in PBR systems?	
	Are you happy with the data quality of the PBR systems?<br>Why/Why not?	

**Section 4: Views and perspectives on MCBR**		
	Can you describe the MCBR system for malaria case reporting?	
	How do you receive and access the data collected by MCBR?	
	How do you analyse the data collected by MCBR? Please give examples.	
	Do you think MCBR is an effective method of malaria surveillance in an elimination setting like Myanmar? Why? If not, why?	
	What are barriers/challenges in managing the MCBR?	
	What are the advantages to using in MCBR system?	
	To what extent does the MCBR improve your access to reporting data?	
	Are you happy with the data quality of the MCBR systems?<br>Why/ Why not?	
	What impact do you perceive that using the MCBR will have on malaria elimination in Myanmar?	
**Section 5: Usability of MCBR**		
	How do you think the MCBR system could be improved?	
	How do you currently use the data from MCBR /DHIS2 to inform decision making around funding/resources allocation?	
	What is the most challenging part of accessing and/or using data gathered through MCBR/DHIS2? Why?	
	What concerns do you have in using/scaling up MCBR? Please elaborate	
	How do you ensure that the data collected by ICMVs is complete and accurate?	
	How is the data collected by MCBR monitored and how are failures reported and addressed?	
**Section 6: Barriers and enablers to applying MCBR**		
	What are the barriers and enablers of implementing and managing the MCBR as a reporting system in the malaria program?	
	Barriers<br>How can we mitigate/solve those barriers?	
	Enablers	

**Section 7: Funding and cost-effectiveness for MCBR**		
	Is MCBR more cost effective than PBR? Why? Why not?	
	How is MCBR funded currently?	
	If MCBR is to be scaled-up and rolled-out nationally, how will it be funded?	
	Do you foresee any potential problems for MCBR funding?	
**Section 8: Supports needed for applying MCBR in malaria program**		
	Which supports are needed for ICMV to expand the MCBR as a routine and effective reporting channel? (technical and training, financial, technological, political, advocacy, administrative, moral etc)<br>Are they been fulfilled?<br>If not, do you see any plan to fulfil those requirements?<br>If not, how can we best fulfil those requirements in time?	
	Which supports are needed for data assistants, M&E staff and/or any data staff who have responsibility to compile, enter and analyse data from MCBR? (technical and training, financial, technological, political, advocacy, administrative, moral etc)<br>Are they been fulfilled?<br>If not, do you see any plan to fulfil those requirements?<br>If not, how can we best fulfil those requirements in time?	
	Which supports are needed for policy makers and/or decision makers to access and utilize data from MCBR? (technical and training, financial, technological, political, advocacy, administrative, moral etc)<br>Are they been fulfilled?<br>If not, do you see any plan to fulfil those requirements?<br>If not, how can we best fulfil those requirements in time?	
**Section 9: Sustainability in the context of ICMV**		
	Do you think MCBR application is sustainable in the context of ICMV if necessary improvements are made and/or necessary supports are provided? Why? Why not?	
	What are the key factors that will determine sustainability of MCBR for malaria reporting and surveillance in the malaria program? How do we address them to sustain the MCBR in long run?	
	Who is responsible for sustaining the MCBR? Do you think NMCP can maintain the operation of MCBR without support from Save the Children? Why? Please discuss in detail including the following discussion points:	

	Server maintenance, server expansion, safeguarding data	
	What kinds of adaptations need to be made to the application and overall system to enable NMCP to take maintain operation of MCBR?	
	What kinds of resources are needed to enable NMCP to maintain operation of MCBR?	
	Can we use app-based reporting channel for reporting of non-malaria ICMV diseases?	
	What kinds of adaptations need to be made to the application and overall system to enable reporting of non-malaria disease covered by ICMV?	
	What kind of resources are needed to enable reporting of non-malaria disease covered by ICMV?	
	Who will be using the app-based reporting channel (BHS, Midwife, etc.)? Please discuss.	
	What kinds of adaptations need to be made to the application and overall system to enable non-ICMV personnel (e.g., BHS, midwives) to use the application?	
	What kind of resources are needed to enable non-ICMV personnel (e.g., BHS, midwives) to use the application?	
**Section 10: Overall**		
	Do you think PBR can be totally replaced by MCBR for reporting of malaria cases in Myanmar malaria elimination program? Why /Why not?<br>If yes, how can we best transition from PBR to MCBR? What are the factors that need to be taken care off in the transition?	
	Do you have any other suggestions/ comments regarding the reporting of malaria cases? If yes, please make comments.	

Do you have any questions for me?

Thank you very much for your participation.

End of session
